# The Reciprocal Association between Problem Gambling and Mental Health Symptoms/Substance Use: Cross-Lagged Path Modelling of Longitudinal Cohort Data

**DOI:** 10.3390/jcm8111888

**Published:** 2019-11-06

**Authors:** Nicki A. Dowling, Carla A. Butera, Stephanie S. Merkouris, George J. Youssef, Simone N. Rodda, Alun C. Jackson

**Affiliations:** 1 Faculty of Health, School of Psychology, Deakin University, Geelong, Victoria 3220, Australia; cabutera@deakin.edu.au (C.A.B.); stephanie.merkouris@deakin.edu.au (S.S.M.); george.youssef@deakin.edu.au (G.J.Y.); s.rodda@auckland.ac.nz (S.N.R.); 2 Melbourne Graduate School of Education, University of Melbourne, Parkville, Victoria 3053, Australia; aluncj@unimelb.edu.au; 3 Centre for Adolescent Health, Murdoch Children’s Research Institute, Royal Children’s Hospital, Parkville, Victoria 3052, Australia; 4 Faculty of Medical and Health Sciences, School of Population Health, University of Auckland, Auckland 1142, New Zealand

**Keywords:** problem gambling, gambling, mental health, substance use, depression, anxiety, alcohol, drug, cross-lagged path models, longitudinal, prospective

## Abstract

To date, studies have highlighted cross-sectional and unidirectional prospective relationships between problem gambling and mental health symptoms or substance use. The current study aims to: (1) examine the reciprocal relationships between problem gambling and mental health symptoms (depression, generalized anxiety)/substance use variables (hazardous alcohol use, daily tobacco use, and drug use) using cross-lagged path models in a prospective general population cohort sample; and (2) determine whether these associations are moderated by age and gender. This study involved secondary data analysis from 1109 respondents who provided data during Wave 2 or 3 (12-months apart) of the Tasmanian Longitudinal Gambling Study (Australia). Depression (odds ratio (OR) = 2.164) and generalized anxiety (OR = 2.300) at Wave 2 were found to have cross-lagged associations with the subsequent development of any-risk gambling (low-risk, moderate-risk, or problem gambling) at Wave 3. Hazardous alcohol use, daily tobacco use, and drug use at Wave 2 were not associated with the development of any-risk gambling at Wave 3. Any-risk gambling at Wave 2 was not associated with the subsequent development of any mental health symptoms or substance use variables at Wave 3. Age and gender failed to be significant moderators in the associations between any-risk gambling and mental health symptoms or substance use variables. Future longitudinal and event-level research is required to further substantiate these prospective relationships, with a view to developing targeted preventions and interventions.

## 1. Introduction

Gambling disorder (formerly pathological gambling) has been re-classified in the Diagnostic and Statistical Manual of Mental Disorders (Fifth Edition) (DSM-5) as an addictive and related disorder alongside substance and alcohol use disorders [1]. In contrast, the term problem gambling is often employed in jurisdictions that employ a public health approach [2] to refer to gambling across a continuum of risk that results in adverse consequences for individuals, families, and communities [3]. In terms of global prevalence rates, in the past year, the standardised prevalence of problem gambling in adults ranged from 0.5% to 7.6% across countries, with an average of 2.3% [4]. Gambling-related harms include financial harm and loss, relationship breakdown, emotional and psychological distress, health decline, cultural harm, reduced work/study performance, and criminal activity, as well as life course and intergenerational harm [5].

### 1.1. Comorbidity of Problem Gambling and Mental Health Disorders

Current evidence suggests that there is a strong comorbidity between problem gambling and mental health issues, with several systematic reviews finding that mood disorders, anxiety disorders, and alcohol and drug dependence are over-represented in both community-representative [6] and treatment-seeking gambling [7] populations. A systematic review of community-representative samples of problem gamblers revealed that the comorbidities with the highest mean prevalence are nicotine dependence (60.1%), any substance use disorder (57.5%), any mood disorder (37.9%), any anxiety disorder (37.4%), alcohol use disorder (28.1%), and illicit drug abuse/dependence (17.2%) [6]. Moreover, there is growing international evidence to suggest that individuals presenting with gambling problems are over-represented amongst alcohol and drug (AOD) services and mental health populations [8,9,10,11,12,13,14,15,16,17,18,19]. For example, a study examining the prevalence of gambling problems in a U.S. representative sample of 3007 respondents reporting past-year treatment for affective disorders revealed conservative lifetime and past-year estimates of 3.1% and 1.4%, respectively, with rates of lifetime problem gambling ranging from 3.1% for depression to 5.4% for social phobia and rates of past-year problem gambling ranging from 0.9% in dysthymia to 2.4% in social phobia [10]. Moreover, systematic review evidence has revealed that the mean prevalence of current or lifetime problem gambling (including gambling disorder) in alcohol and substance use treatment services is 22.8% [19]. 

### 1.2. The Temporal Relationship between Problem Gambling and Mental Health Disorders

Although the findings from these studies suggest that problem gambling is comorbid with many mental health disorders, their cross-sectional nature precludes an explication of the temporal order between these disorders. The findings of age of onset studies using retrospective data [20,21,22] suggest for most respondents that anxiety disorders, mood disorders, and alcohol and other drug use disorders typically predate the onset of problem gambling. These studies suggest that the only exceptions may be nicotine dependence, and to a lesser extent, post-traumatic stress disorder, major depressive disorder, and phobias. Parallel survival analyses using this data [22] reveal that although there are significant time-lagged predictive associations for problem gambling predicting the subsequent onset of some mental health disorders (bipolar disorder, phobia, post-traumatic stress disorder, alcohol or other drug dependence, and nicotine dependence), there are many more associations for other disorders predicting the subsequent onset of problem gambling. 

Although retrospective recall of age of onset is helpful in exploring the possible temporal or causal relationships between problem gambling and comorbid mental health conditions, it is limited by a reliance on retrospective study designs that may introduce recall and reporting biases. Moreover, the issue of causality is confounded in these studies by the fact that some disorders naturally have an earlier age of onset and that gambling in most jurisdictions is illegal for adolescents. There is, however, an emerging literature of prospective and longitudinal research on the determinants of problem gambling. A systematic review and meta-analysis exploring the early risk and protective factors (measured in childhood, adolescence, or young adulthood) that are longitudinally associated with the development of gambling problems [23] revealed that depressive symptoms, alcohol use frequency, cannabis use, illicit drug use, and tobacco use, but not anxiety symptoms, were positively associated with subsequent problem gambling, with small but significant effect sizes. A smaller literature employing adult samples from the general population also generally suggests that depressive symptoms [24,25,26,27] and alcohol and other substance use disorders [24,26,27,28,29,30] predict the subsequent development of at-risk or problem gambling over a one- to ten-year time period. These studies, however, also generally identify positive associations between anxiety symptoms and the subsequent development of gambling problems [24,25,26,27,29] over a one- to five-year time period.

There is also limited evidence that problem gambling is a risk factor for the subsequent occurrence of mental health disorders. Most longitudinal studies in youth [31] and adult [32,33,34,35] cohorts have consistently found that problem gambling is associated with an increased likelihood of subsequent mood disorders (such as major depressive episodes, major depressive disorder, dysthymia, bipolar disorder, mania, hypomanic episodes), anxiety disorders (such as panic disorder, specific phobia, social phobia, PTSD, generalized anxiety disorder), and alcohol and other drug use disorders (such as alcohol use disorder, alcohol dependence, cannabis and other illegal drug use, other drug dependence, nicotine dependence) across follow-up periods between two and five years. Although these associations are evident after adjusting for socio-demographic characteristics [31,32,33] some, but not all, are attenuated after controlling for psychiatric comorbidity, health behaviours, physical health, medical conditions, health-related quality of life, and stressful life events [32,34,35].

### 1.3. Reciprocal Relationships between Problem Gambling and Psychiatric Disorders

Taken together, the findings of the available literature suggest that the clear majority of mood, anxiety, and alcohol and other drug use disorders typically predate and predict the onset of problem gambling. However, problem gambling also appears to be a risk factor for the development of these disorders, although some, but not all, of these associations are attenuated after controlling for other psychiatric, health, and medical factors. Interestingly, however, few studies to date have investigated reciprocal prospective relationships between problem gambling and psychiatric disorders. 

Several studies have explored these relationships using growth curve modelling using longitudinal data. Using prospective data from four waves (12- to 18-month intervals) of the Manitoba Longitudinal Study of Young Adults (MLSYA), Chinneck and colleagues [36] examined whether the relationship between problem gambling and depression is directional (with one reliably preceding the other), bidirectional, or pathoplastic (whereby increases in one disorder result in increases in the other over time). Bivariate growth curves revealed that the disorders were positively correlated with each other at Waves 1, 2, and 4 but that neither disorder was a risk factor for the other and that they were not pathoplastically related. Similarly, using four waves of data (nine to 13 months apart) from the Leisure, Lifestyle, and Lifecycle Project in Alberta, Canada, across five years, Mutti-Packer and colleagues [37] examined the temporal associations between problem gambling and alcohol misuse from adolescence (ages 13 to 16) to young adulthood (ages 17 to 21) using parallel-process latent growth curve modelling. An unconditional parallel process model (no covariates added) indicated that baseline levels of problem gambling symptoms were not associated with change over time in alcohol misuse; but that higher baseline levels of alcohol misuse were associated with steeper declines in problem gambling over time. However, when covariates (sex, parental household income, smoking status, and illicit drug use) were added to the model, the evidence for a relationship diminished suggesting that changes in one outcome were not related with changes in the other.

Other studies have examined possible reciprocal relations between problem gambling and mental health symptoms using cross-lagged analyses. These analyses are stringent, in that they control for stability in each construct over time and a cross-lagged effect only occurs when a variable predicts longitudinally above stability in the outcome variable. Wanner and colleagues [38] employed two community male samples to explore the cross-lagged links (the prospective links of one problem behaviour to another problem behaviour) among multiple problem behaviours (problem gambling, substance use, theft, and violence) from mid-adolescence (age 16) to young adulthood (age 23). After controlling for concurrent links and shared variance among the variables, the results of this study revealed that gambling problems were not longitudinally associated with substance use and that substance use was not longitudinally associated with gambling problems. Similarly, using one of the same samples, Dussault and colleagues [39] employed cross-lagged structural equation modelling to investigate the degree to which common antecedent factors (socio-family risk and impulsivity) explain the prospective links between depressive symptoms and gambling problems from late adolescence (age 17) to early adulthood (age 23). Results revealed that gambling problems at age 17 predicted an increase in depressive symptoms from age 17 to age 23, and that depressive symptoms at age 17 predicted an increase in gambling problems from age 17 to age 23.

### 1.4. Study Aims

Although these studies investigating reciprocal relationships have employed methodologies controlling for baseline symptoms of problem gambling and mental health comorbidities, their findings may be specific to developmental age (adolescence and young adulthood) and may therefore not be generalizable to adult samples. To date, there have been no studies that have investigated reciprocal links between problem gambling and mental health or substance use conditions using a prospective longitudinal design in a general population adult sample. The current study aimed to expand on existing research by examining reciprocal relationships between problem gambling and multiple mental health symptoms (depression, generalized anxiety)/substance use variables (hazardous alcohol use, daily tobacco use, and drug use) using cross-lagged path models in a prospective adult cohort sample. A secondary aim was to determine whether these associations are moderated by age and gender.

## 2. Experimental Section

### 2.1. Participants

The current study involves secondary data analysis of a community sample of respondents who participated in the Tasmanian Longitudinal Gambling Study in Australia, which was a component of the Third Social and Economic Impact Study (SEIS) of Gambling in Tasmania [40]. Respondents in the Tasmanian Longitudinal Gambling study are a sub-sample of respondents from the Second SEIS of Gambling in Tasmania (Wave 1) [41]. Only data from Waves 2 and 3 of the Tasmanian Longitudinal Study were employed in this study as mental health variables were not collected in Wave 1. The final sample consisted of 1109 respondents who provided data for either Wave 2 or Wave 3 of the Tasmanian Longitudinal Gambling Study. The mean age of respondents in this final sample was 59.09 years (standard deviation (SD) = 14.99) and 62.4% were male. 

### 2.2. Measures

Problem gambling severity, mental health symptoms (depression, generalized anxiety), and substance use variables (hazardous alcohol use, daily tobacco use, and drug use) were measured in Waves 2 and 3 of the Tasmanian Longitudinal Gambling Study. 

Problem gambling severity. Past-year problem gambling severity was assessed using the Problem Gambling Severity Index (PGSI) of the Canadian Problem Gambling Index (CPGI) [42]. The PGSI consists of nine items that are rated on 4-point scale, with response options ranging from never (0) to almost always (3). Scores range from 0 to 27 and can be used to categorise gambling severity across the continuum of risk: non-problem gambling (scores of 0), low-risk gambling (scores of 1–2), moderate-risk gambling (scores of 3–7), and problem gambling (scores of 8 or more). The PGSI has shown very good internal consistency, validity, sensitivity, and specificity in previous research [42].

Depression. Depression was assessed using the Patient Health Questionnaire-2 (PHQ-2) [43], which is a 2-item questionnaire rated on a 4-point scale. The PHQ-2 rates the presence of depressive symptoms from not at all (0) to nearly every day (3). This screening instrument comprises the first two items of the Patient Health Questionnaire and represents the core DSM-IV items for major depressive disorder (MDD). A score of 3 or greater indicates a positive screen for MDD [43]. The PHQ-2 has displayed good sensitivity (0.83) and specificity (0.90) for classifying MDD [43]. 

Generalized anxiety. Generalized anxiety was assessed using the Generalized Anxiety Disorder-2 (GAD-2) [44], which is a 2-item questionnaire rated on a 4-point scale. The GAD-2 rates the presence of anxiety-related symptoms from not at all (0) to nearly every day (3). This screening instrument consists of the first two items of the Generalized Anxiety Disorder questionnaire and represents the core DSM-IV items for Generalized Anxiety Disorder. A score of 3 or greater indicates a positive screen for GAD [44]. The GAD-2 has displayed good sensitivity (0.86) and specificity (0.83) in detecting Generalized Anxiety Disorder [44]. 

Alcohol use. Hazardous alcohol use was assessed using the Alcohol Use Disorder Identification Test—Consumption (AUDIT-C) [45]. The AUDIT-C comprises the first three items of the 10-item AUDIT that measure alcohol consumption on a 5-point scale with various response options. A score of 4 or more for men (sensitivity = 0.91, specificity = 0.70) is considered optimal for identifying heavy drinking [45]. In addition, a score of 4 or more for men (sensitivity = 0.86, specificity = 0.72) and 3 or more for women (sensitivity = 0.60, specificity = 0.96) is considered optimal for identifying hazardous/heavy drinking and/or active alcohol use disorder [45,46].

Tobacco use and drug use. Two separate items were used to measure past-year tobacco use and drug use (including illicit drug use and prescription medication misuse). These items were based on the single-item screening test for drug use in primary care [47]. This screening test measures frequency of use in the past year, where a response of at least once in the past year is considered positive for drug use. This single item has demonstrated excellent sensitivity (0.86–0.96) and specificity (0.89–0.96) in detecting past year drug use [47]. The response options applied for these items in this study were: every day; 4–6 times a week; 2–3 times a week; once a week; 2–3 times a month, monthly or less; and not in the last year/never. 

### 2.3. Procedure

The first wave of the Tasmanian Longitudinal Gambling Survey comprised a sub-sample of 2027 respondents from the second Tasmanian SEIS of Gambling in Tasmania. The second SEIS of Gambling in Tasmania [41], which was conducted between February 7 and March 3, 2011, involved Computer-Assisted Telephone Interviews (CATI) of a stratified random sample of 4303 adult respondents in Tasmania using a random digit dialling and exchange-based telephone survey of registered landline telephone numbers. A disproportionate stratified sample design was employed in which selected local government areas (LGAs) of high and low electronic gaming machine (EGM) density and high and low socio-economic status were over-sampled relative to their population. The overall survey participation rate (defined as the number of completed interviews divided by the sum of the completed interviews plus refusals) was 48.8% and the average interview length was 15.8 min. A sub-sample of 2027 respondents (47% of the overall sample) was administered as a supplementary survey in which they were asked questions relating to psychosocial issues. This sub-sample comprised all low-risk, moderate-risk, and problem gamblers (PGSI score >0), all past-year EGM gamblers, a randomly selected one-third of non-gamblers (no past-year gambling participation), and a randomly selected one-third of non-problem gamblers (past-year gambling participation and PGSI score = 0). For further methodological details, see [41].

Wave 2 took place over the period November 6 to December 22, 2013 (2 years and 9 months after the Wave 1 survey). The in-scope sample for this survey was respondents who were administered the main and supplementary surveys in the second SEIS (Wave 1) and who agreed to be re-contacted (*n* = 1879). Of these, 100 were employed for the pilot, 223 were unusable (e.g., disconnected, not a residential number, fax/modem, incoming call restrictions), 186 were uncontactable (e.g., answering machine, no answer), 167 were out of scope (e.g., passed away, too old/frail/hearing impaired to complete survey, denied previous participation, language other than English, named person not known), 72 were unresolved contacts (e.g., appointment, away from home), 85 refused, and 7 terminated the survey midway. The total achieved sample size for the Wave 2 survey was therefore 1039. The consent rate was 82.1%, which represents the number of completed interviews as a percentage of the number of in-scope people actually contacted. The average interview length was 24.2 min. 

Wave 3 of the survey took place over the period November 19 to December 21, 2014 (approximately one year after the Wave 2 survey). The in-scope sample for this survey was respondents to Wave 2 who agreed to be recontacted, and those who were unable to be interviewed in Wave 2 but remained a valid contact (*n* = 1269). Of these, 81 were unusable (e.g., disconnected, fax/modem, incoming call restrictions), 165 were uncontactable (e.g., answering machine, no answer, maximum non-contact all attempts), 52 were out of scope (e.g., passed away, too old/frail/hearing impaired to complete survey, denied previous participation, named person not known), 51 were unresolved contacts (e.g., appointment, away from home), 91 refused, and 9 terminated the survey midway. The total achieved sample size for the Wave 3 survey was therefore 820. The consent rate was 84.4%, which represents the number of completed interviews as a percentage of the number of in-scope people actually contacted. The average interview length was 26.2 min. The average interview lengths were longer for Waves 2 and 3 of the study than Wave 1 as no sub-sampling procedures were employed in these waves. Weights were generated for the Waves 2 and 3 survey data using raking procedures using benchmarks based on Wave 1. For further methodological details, see [40].

Ethics approval was originally obtained by the University of Melbourne’s Humanities and Applied Sciences Research Ethics Committee (Approval number: 1340411.3 and an ethics amendment application was approved by the Deakin University Human Research Ethics Committee.

### 2.4. Statistical Analyses

All data cleaning, basic analyses, and missing data methods were conducted in Stata 13 [48]. Respondents who did and did not complete Wave 2 and Wave 3 were compared on key Wave 1 variables, including age, gender, PGSI problem gambling severity, and past-year gambling participation. There was a significant difference between respondents who completed Wave 2 and Wave 3 and respondents who did not complete Wave 2 and Wave 3 on age. At Wave 2, non-completers were younger (M (mean) = 51.37, SD = 18.72) than the remaining sample (M = 53.36, SD = 14.74, *p* < 0.001). Similarly, non-completers were younger (M = 52.61, SD = 18.13) than the remaining sample at Wave 3 (M = 56.10, SD = 14.73, *p* < 0.001). However, no significant differences were identified for gender (Wave 2: *p* = 0.160; Wave 3: *p* = 0.141), PGSI problem gambling severity (Wave 2: *p* = 0.807; Wave 3: *p* = 0.956), or past-year gambling participation (Wave 2: *p* = 0.224; Wave 3 *p* = 0.494). To account for missing data in Wave 2 and Wave 3, we used the method of multiple imputation by chained equations, which is considered one of the best practice approaches to deal with missing data [49]. Specifically, each variable was imputed using a chained approach where regression models were specified for each variable as a logistic model, except for PGSI which was ordinal logistic and age which was linear regression. All estimates reported were pooled over 50 datasets using Rubin’s rules [50]. Before imputation, all variables used in analysis were binary coded due to all variables exhibiting very strong positive skew and high zero counts. Specifically, as there were low rates of respondents classified in the problem gambling category (PGSI >8), all categories of problematic gambling on the PGSI (low-risk gambling, moderate-risk gambling, and problem gambling) were combined to create a binary variable representing any-risk gambling. Mental health symptoms and substance use variables were binary coded according to recommended clinical cut-off scores: scores of 3+ on the PHQ-2 [43] and GAD-2 [44] and scores of 4+ for males and 3+ for females on the AUDIT-C [45], a response of every day on the single-question screening test for drug use for tobacco use (i.e., daily tobacco use); and a response of at least once in the past year on the single-item screening test for drug use [47].

To address the primary aim, a series of cross-lagged, logistic regression models were completed using Mplus Version 7.2 [51]. A separate analysis was conducted for each of the mental health or substance use variables, which resulted in five cross-lagged path models in total. Specifically, in each of the cross-lagged models, any-risk gambling at Wave 3 was regressed on to both at-risk gambling and mental health symptoms at Wave 2. Simultaneously, each mental health symptom or substance use variable at Wave 3 was regressed on to both at-risk gambling and mental health symptoms or substance use variables at Wave 2. Socio-demographic variables (age and gender) were adjusted for in each of the cross-lagged models. This cross-lagged approach therefore allowed examination of both autoregressive and reciprocal relationships between any-risk gambling and mental health symptoms or substance use variables over time. A second series of analyses systematically examined whether these cross-lagged relationships were moderated by age (continuous variable) then gender (adjusting for gender in the age-moderated regression analyses and age in the gender-moderated regression analyses). For each of these analyses, two additional interaction terms were included as predictors in each model (e.g., age × any-risk gambling and age × mental health/substance use variable).

## 3. Results

Descriptive statistics for each of the variables of interest are represented in Table 1. After imputation, nearly 10% of the sample were classified as any-risk gamblers (i.e., classified in the low-risk, moderate-risk, and problem gambling categories) on the PGSI [42] at both waves. Across the two waves, the sample was most likely to report hazardous alcohol use (57.9–58.8%), followed by daily tobacco use (13.3–14.6%), generalized anxiety (13.4–13.6%), and depression (10.1–12.3%). Smaller proportions of respondents reported drug use (4.8–5.5%). 

**Figure 1:**
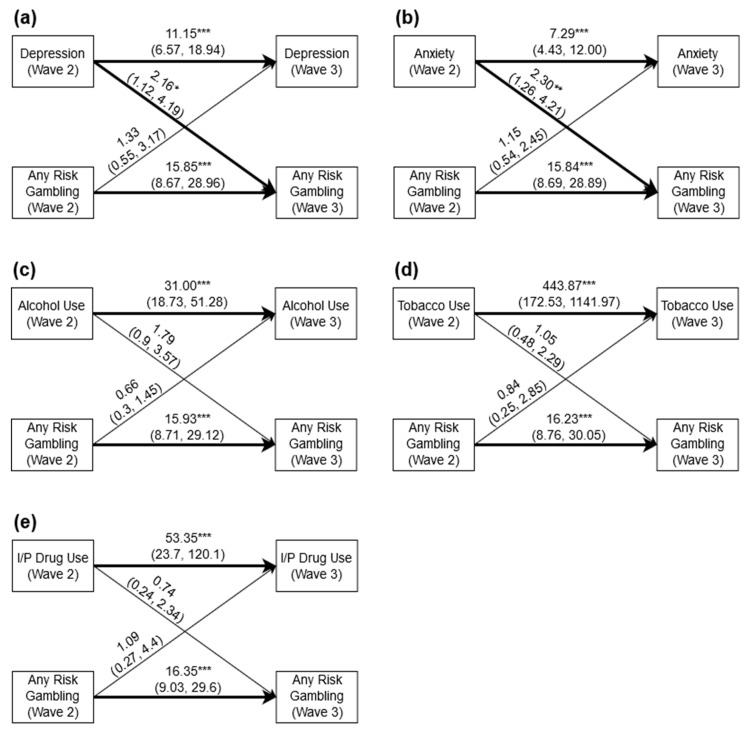
(**a**) Cross-lagged associations between any-risk gambling and depression; (**b**) cross-lagged associations between any-risk gambling and generalized anxiety; (**c**) cross-lagged associations between any-risk gambling and hazardous alcohol use; (**d**) cross-lagged associations between any-risk gambling and daily tobacco use; (**e**) cross-lagged associations between any-risk gambling and drug use. Estimates = odds ratios (95% confidence intervals); I/P = illicit and prescription; * *p* < 0.05; ** *p* < 0.01; *** *p* < 0.001.

Figure 1 displays the pooled cross-lagged associations between any-risk gambling and mental health symptoms (depression, generalized anxiety) or substance use variables (hazardous alcohol use, daily tobacco use, and drug use). There were strong autoregressive relationships for all mental health symptoms/substance use variables and any-risk gambling. For example, any-risk gambling at Wave 2 predicted any-risk gambling at Wave 3 across all models (odds ratio (OR) = 15.847–16.346). Similarly, depression at Wave 2 predicted depression at Wave 3 (OR = 11.156; Figure 1a), generalized anxiety at Wave 2 predicted generalized anxiety at Wave 3 (OR = 7.286; Figure 1b), hazardous alcohol use at Wave 2 predicted hazardous alcohol use at Wave 3 (OR = 31.000; Figure 1c), daily tobacco use at Wave 2 predicted daily tobacco use at Wave 3 (OR = 444.077; Figure 1d), and drug use at Wave 2 predicted drug use at Wave 3 (OR = 53.356; Figure 1e).

With respect to cross-lagged relationships, depression (OR = 2.164; Figure 1a) and generalized anxiety (OR = 2.300; Figure 1b) at Wave 2 predicted any-risk gambling at Wave 3. There was, however, no evidence of a cross-lagged association between any-risk gambling at Wave 2 and depression or generalized anxiety at Wave 3. There were also no cross-lagged relationships between hazardous alcohol use (Figure 1c), daily tobacco use (Figure 1d), or drug use (Figure 1e) at Wave 2 and any-risk gambling at Wave 3; nor between any-risk gambling at Wave 2 and hazardous alcohol use (Figure 1c), daily tobacco use (Figure 1d), or drug use (Figure 1e) at Wave 3. All cross-lagged paths were examined for moderation by age and gender; however, no interaction effects were identified for age or gender in any of the analyses. 

## 4. Discussion

The current study is the first to employ a cross-lagged study design to examine the reciprocal longitudinal associations between problem gambling and mental health symptoms (depression, generalized anxiety) or substance use variables (hazardous alcohol use, daily tobacco use, and drug use) in a general population sample. Overall, the findings revealed that depression and generalized anxiety at Wave 2, but not hazardous alcohol use, daily tobacco use, or drug use, had cross-lagged associations with subsequent any-risk gambling at Wave 3. Moreover, any-risk gambling at Wave 2 did not have any cross-lagged associations with the subsequent development of any of the mental health symptoms or substance use variables at Wave 3. Overall, these findings are generally consistent with the available literature that suggests that the majority of mood, anxiety, and alcohol and other drug use disorders typically predate and predict the onset of problem gambling, but that many of the associations between problem gambling and the subsequent development of mental health symptoms are attenuated after controlling for other factors. 

### 4.1. Cross-lagged Associations between Wave 2 Mental Health Symptoms/substance use Variables and Wave 3 Any-Risk Gambling

In this study, only depression and generalized anxiety at Wave 2 had cross-lagged associations with any-risk gambling at Wave 3. The finding relating to depression is consistent with those from both youth cohort studies [23] and general population cohort studies [24,25,26,27], whereby depressive symptoms and mood disorders consistently predict the subsequent development of at-risk or problem gambling. While the finding relating to generalized anxiety is not consistent with the systematic review conducted in youth cohort studies [23], it is generally consistent with the small body of research conducted in adult cohort studies [24,25,26,27,29]. While it may be tempting to surmise that this relationship may only hold for adult samples, it is important to note that the systematic review results are based on a very small number of studies investigating this relationship in youth (k = 3 with 4 associations) [23]. Moreover, there are other methodological differences between the youth and adult cohort studies, apart from the age of the cohort, including the size of the samples, the measures of anxiety employed, the measures of problem gambling employed, the length of the follow-up period, and the gender composition of the samples [23]. Nevertheless, on the basis of the available evidence, it does appear that, at least in adults, internalising symptoms may precede the development of gambling problems, implying that gambling is used as a way to regulate negative aversive emotional states [39,52,53]. 

There were no cross-lagged associations between any substance use variable (hazardous alcohol use, daily tobacco use, and drug use) at Wave 2 and subsequent any-risk gambling at Wave 3. These findings are inconsistent with the majority of longitudinal studies, in which there is little heterogeneity in effect size estimates between the associations for problem gambling and alcohol- and drug-related variables, at least in youth cohort studies [23]. There are, however, some equivocal results relating to all of the indices of alcohol and drug use under investigation across the youth and adult literatures [24,26,27,30,38,54,55,56]. This is particularly the case for female samples [56] and after controlling for gambling problems and drug-related variables at the initial time-point (e.g., [37,38]). Moreover, the effect sizes for associations between alcohol- and drug-related variables and subsequent gambling problems are generally very small [23]. It is worth noting that the stability (autoregressive) coefficients are larger for substance use variables (ORs = 31.00–443.87) than the mental health symptoms (ORs = 7.29–11.15), which makes it more difficult to observe a cross-lagged effect for the substance use variables. It may be, however, that a longitudinal association between alcohol- and drug-related variables and subsequent gambling problems exists in only a sub-sample of problem gamblers. Such an effect may be “washed out” when using the estimates from the full sample [23,57]. Future prospective research in large longitudinal community-representative samples or using person-centred methods, such as latent class analysis or event-related approaches, may help to elucidate the exact nature of these relationships. 

### 4.2. Cross-Lagged Associations between Wave 2 Any-Risk Gambling and Wave 3 Mental Health Symptoms/Substance Use Variables

Similarly, any-risk gambling in Wave 2 did not have cross-lagged associations with any of the mental health symptoms or substance use variables in Wave 3. These findings generally contrast with those from the small available literature in both youth and adult samples which consistently shows longitudinal associations between problem gambling and the development of mental health or substance use symptoms [31,32,33,34,35,39]. However, there is considerably less research available that examines the prospective relationship between problem gambling and the subsequent development of mental health or substance use symptoms than that examining the prospective relationship between mental health symptoms and substance use and the subsequent development of problem gambling. Moreover, there are some inconsistencies in this literature, with equivocal findings in relation to the indices of mental health symptoms or substance use variables under investigation [32,33,34,35,37,38]. The failure to identify significant findings in the context of a cross-lagged design in which any-risk gambling and mental health symptoms or substance use variables at the initial time-point are adjusted for is consistent with previous findings that some prospective associations are attenuated after controlling for other factors [32,34,35,36,37,38]. Again, however, it may be that the effects from a subgroup of respondents with mental health symptoms or substance use variables were washed out in the estimates from the full sample [23,57].

### 4.3. Age and Gender as Moderators in the Associations between Any-Risk Gambling and Mental Health Symptoms/Substance Use Variables

Finally, age and gender failed to be significant moderators in the associations between any-risk gambling and mental health symptoms or substance use variables. There is considerable evidence of sex-specific patterns in the relationship between problem gambling and mental health symptoms or substance use variables. Depression and anxiety symptoms are often more strongly associated with female problem gambling than male problem gambling [35,58,59,60,61,62], while hazardous alcohol use, tobacco use, and drug use are more likely to be associated with male problem gambling than female problem gambling [35,60,62,63]. Few studies, however, have employed sex as a moderator of the relationship between psychiatric factors and problem gambling. Moreover, the limited evidence suggests that sex often fails to statistically moderate these relationships. For example, there is evidence that sex does not buffer or exacerbate the relationships between problem gambling and mood or anxiety problems, alcohol use problems, nicotine dependence, or substance use disorders [61,64,65]. Even fewer studies examine the associations between gambling and mental health measures stratified by age [66,67] or the moderating effects of age in predicting problem gambling [67]. The current findings suggest that the associations between at-risk gambling and mental health factors are similar across both sexes and age.

### 4.4. Study Limitations

To date, the majority of longitudinal gambling research has been conducted in youth cohort study samples, the findings of which cannot be generalized to the wider community [23]. Moreover, many of the available studies fail to measure gambling problems at the first evaluation period, which not only precludes the drawing of conclusions regarding changes in problem gambling status across time, but also precludes the examination of cross-lagged links among problem gambling and other factors [23]. The current research expands on this currently available literature by being the first to examine cross-lagged links between gambling problems and mental health symptoms/substance use variables in a large general population sample, as well as controlling for socio-demographic characteristics. 

The reported findings must, however, be considered in light of some limitations. First, because the sample size was too small to capture a sufficient number of adults with gambling problems, a low threshold definition of problem gambling in all analyses, including those at lower levels of risk, was employed. This precluded the examination of the cross-lagged associations across the continuum of gambling risk and may have reduced the comparability to existing research on problem gambling. Even using this procedure, the relatively small numbers of any-risk gamblers in this study may have limited statistical power and contributed to the relatively small effect sizes identified. Second, the current research employed Waves 2 and 3 of the Tasmanian Longitudinal Gambling Study due to the collection of mental health and substance use variables only across these waves, which reduced the longitudinal time period to one year, which is at the lower end of the currently available literature. Third, as with most epidemiological studies, data were collected from respondents using brief self-report screening instruments for mental health and substance use issues, which precludes the ability to make formal psychiatric diagnoses. Moreover, the mental health measures employed in this study may not represent mood and anxiety disorders more generally as they evaluated very specific internalising disorder symptoms (major depression and generalized anxiety). Fourth, it remains unclear as to whether the major depression and generalized anxiety symptoms are independent longitudinal predictors of escalations in any-risk gambling over time as they are highly overlapping constructs and the cross-lagged analyses for each did not control for the other. Fifth, the current study consisted of a relatively older adult sample, which may limit the generalizability of the findings. Finally, the respondents not contacted at follow-up evaluations may have displayed higher rates of problem gambling and other pathological outcomes relative to their successfully contacted counterparts [68,69]. 

Future research in larger general population samples with multiple waves of data across longer follow-up periods and the use of semi-structured diagnostic interviews is required to further elucidate the nature of these cross-lagged relationships. Future research with larger samples would have the advantage of being able to explore the degree to which low- and moderate-risk gambling are associated with mental health symptoms or substance use variables. In combination with diagnostic instruments of mental health and substance use, they may also allow for the elucidation of the dimensions of depression and anxiety (e.g., somatic, affective, cognitive, and behavioural) that are associated with the subsequent development of gambling problems, which has the potential to inform treatment approaches. Further prospective naturalistic research at the event level would also enhance our understanding of the interaction between mental health symptoms or substance use and gambling episodes as they occur in real life.

### 4.5. Implications for Research Translation

These limitations notwithstanding, the findings of the current study offer important insights regarding the prospective and reciprocal relationships between gambling problems and mental health symptoms or substance use variables. Accurately identifying modifiable characteristics that can be targeted to lower future risks for gambling problems and vice versa is necessary for the development of effective prevention and intervention initiatives. The study findings suggest that depression and generalized anxiety symptoms appear to have some aetiological role in the development of gambling problems, indicating a need for these psychiatric conditions to be identified and effectively treated. They suggest that gambling treatment providers routinely screen for co-occurring depression and anxiety symptoms in people seeking treatment for gambling problems and deliver tailored treatment plans for those screening positive for co-occurring gambling problems. This is particularly important, given evidence that depression and anxiety comorbidity in problem gambling is associated with more complex clinical presentations [70,71] and can negatively influence the outcomes of treatment [70,72,73,74,75]. Likewise, prevention efforts may also be enhanced by mental health service providers routinely screening for gambling problems and provide appropriate resources and referrals. The findings of this study suggest that depression and generalized anxiety are related to gambling problems across the continuum of risk (i.e., any-risk gambling), suggesting that screening for both problem gambling and at-risk gambling in these services may enhance prevention efforts. A recent systematic review has found that several brief screening instruments display satisfactory classification accuracy in detecting both problem and at-risk gambling: Brief Problem Gambling Screen (BPGS-2), NORC DSM-IV Screen for Gambling Problems including Loss of Control, Lying, and Preoccupation items (NODS-CLiP), Problem Gambling Severity Index-Short Form, NODS including Preoccupation, Escape, Risked Relationships, and Chasing items (NODS-PERC), and NODS-CLiP2 [76].

## 5. Conclusions

The present community-based longitudinal investigation highlights the significance of mental health symptoms such as depression and generalized anxiety in the progression and development of gambling problems. However, the lack of cross-lagged associations in relation to alcohol and drug use indicates that further research is required to elucidate the nature of these relationships. Highlighting the temporal associations between mental health symptoms or substance use and gambling problems provides important information for the development of treatment, intervention, and prevention programs. It also supports the necessity for clinicians to screen for both disorders when individuals present to health care systems. Future longitudinal and event-level research should endeavour to further substantiate these relationships for the purposes of developing targeted and specific interventions. 

## Figures and Tables

**Table 1:** Pooled proportions and their 95% confidence intervals (CI) ^a.^

	Wave 2% (95% CI)	Wave 3% (95% CI)
PGSI Any-risk gambling	9.5 (7.7–11.3)	9.7 (7.7–11.7)
PHQ-2 Depression	12.3 (10.3–14.3)	10.1 (8.0–12.3)
GAD-2 Generalized anxiety	13.4 (11.4–15.5)	13.6 (11.2–16.0)
AUDIT-C Hazardous alcohol use	58.8 (55.3–62.3)	57.9 (54.2–61.6)
Daily tobacco use	13.3 (11.2–15.3)	14.6 (12.4–16.8)
Drug use	4.8 (3.5–6.1)	5.5 (3.9–7.0)

^a^ After multiple imputation. PGSI: Problem Gambling Severity Index; PHQ-2: Patient Health Questionnaire-2; GAD-2: Generalized Anxiety Disorder-2; AUDIT-C: Alcohol Use Disorder Identification Test—Consumption.
